# Out-Look on Worldwide Trends of Related Studies on Citrus Waste as Feed for Livestock Production: A Scientometric Analysis

**DOI:** 10.3389/frma.2022.869974

**Published:** 2022-04-22

**Authors:** Emrobowansan Monday Idamokoro, Yiseyon Sunday Hosu

**Affiliations:** ^1^Small-Scale Agribusiness and Rural Non-farm Enterprise, Niche Area, Walter Sisulu University, Mthatha, South Africa; ^2^Department of Economics and Business Sciences, Faculty of Commerce and Administration, Walter Sisulu University, Mthatha, South Africa

**Keywords:** citrus, bibliometric evaluation, alternative feed resource, livestock production, bibliometrics, feed, livestock, alternative resource

## Abstract

The present study aimed to reveal the abundant tapestry of research on citrus waste and livestock feed, taking into account the recurring challenges posed by feed shortage and high price of conventional animal feed in livestock farming. In total, 565 articles were retrieved in a BibTeX format for analysis using bibliometric package in R studio. The retrieved data included, but not restricted to authors, citations, keywords, journals, and institutions. Published outputs on citrus waste and animal feed for livestock production obtained from Scopus and web of science (WOS) databases were used in this study. The field of citrus waste and livestock feed research experienced an increase in terms of research outputs with an annual growth of 10.20% during the study period. Based on the country level, Brazil was rated first with an aggregate sum of publications (*n* = 81), with China having a huge global academic influence with most top article citations (*n* = 1,338). The topmost authors' keywords commonly used in the studied research area were citrus pulp (*n* = 48), pectin (*n* = 26), performance (*n* = 22), and citrus (*n* = 33), which created a hint on associated studies on citrus waste and livestock feed. The present study provides a global trend to traverse the intellectual quandary on citrus waste and livestock feed research, and guidance for further studies in this field. It is essential to stress that the present study only dealt with core areas of citrus waste and livestock feed research, hence, it is anticipated that new empirical research and prospective solutions would afford new knowledge insight on citrus waste and livestock feed as new studies evolve.

## Introduction

Shortage of good quality livestock feeds required to sustain livestock production and performance, especially during drought or dry seasons has been reported to affect the advancement of animal production in most countries and especially in developing countries. Non-conventional feed resources including crop residues are in regular search to lower the over-reliance on conventional feedstuff resources and to reduce feeding costs during animal production. The moderately high prices of livestock feed in many countries are part of the identifiable challenges in animal production among others (Basir and Toghyani, 2017). Livestock feeding systems based on naturally abundant by-product feedstuffs (BPFs) are often a practical alternative for animals because their gut microbial ecosystem can utilize BPFs, which often contain high levels of structural fiber to meet their nutrient requirements for growth, maintenance, production, and reproduction performance (Bombik et al., 2002; Hernandez et al., 2004; Yang et al., 2009).

Wastes from fruits and/or vegetables produced in crop farms or those gotten after processing from agro-allied industries, are, however, well-endowed in some vital nutrients that have the potential to be supplemented in livestock diets as feedstuffs. The incorporation of fruits wastes in animal feeds has been found to enhance palatability of diet and consequently increase feed consumption in addition to decreasing the cost of the feed (Chaudry et al., 2004). Most livestock farmers of late fancy the use of alternative feedstuff resources which are proved to be nutritious and beneficial for their animals and have been certified to have no harmful effects for human consumption (Ellin-Doyle, 2001).

One of such naturally abundant fruit crops that can be explored in most countries globally as a feedstuff for livestock production is citrus (Pourhossein et al., 2015; Alnaimy et al., 2017; Seidavi et al., 2020). Citrus fruits are of various varieties, including lemons, limes, oranges, tangerines, and mandarins among others. The production of citrus is increasing day by day because of increasing consumer demands. Oranges, a part of citrus fruit accounts for more than 60% of the world's citrus production with ~82 million tons (Alnaimy et al., 2017). Citrus farms and citrus agro-allied processing industries generate huge amounts of citrus wastes yearly. According to Sharma et al. (2017), citrus peel waste alone gives rise to nearly 50% of the wet fruit mass. Citrus waste can be harvested globally on a massive and large scale of land giving rise to ~50–60% total of its production level and industrial significance (Satari and Karimi, 2018). Citrus fruit waste is of enormous economic worth as it contains plenty of various useful nutritional compounds, including carotenoids, flavonoids, dietary fiber, essential oils, sugars, polyphenols, and ascorbic acid, as well as substantial amounts of some trace elements, which can benefit livestock.

Furthermore, the citrus waste contains high levels of sugars suitable for fermentation for bioethanol production (Varmie and Mamta Thakur, 2021). It is well-established that citrus fruits and citrus products are rich sources of vitamins, minerals, and dietary fibers (non-starch polysaccharides) essential for the nutrition, growth, and overall development of animals (Economos and Clay, 1999; Varmie and Mamta Thakur, 2021). Recently, citrus fruits have been observed for other non-nutrient yet biologically active compounds such as flavonoids, carotenoids, vitamins, and minerals which could be medicinal for animals (Benavente-Garcia and Castillo, 2008).

Citrus waste is partly the unwanted/un-used materials (pulp, peels) obtained from farms and agro-allied industries after processing. However, the waste from citrus may cause several potential economic and environmental problems because of its fermentability if not properly converted to environmentally friendly value-added products (Tripodo et al., 2004). The current volume of citrus waste often gotten as by-products from citrus farms and other agro-allied processing industries could be useful in strengthening the nutritional gap experienced in animal feeds and further evading the age-long competition for edible grains consumption (e.g., maize) often created between human and livestock. Therefore, the utilization of citrus waste as animal feeds to advance livestock farming needs more attention. To the best of our knowledge, and based on the information that we can gather, no published article has analyzed scientific pieces of literature on scientometric studies on the current research trends on citrus waste as animal feed for livestock production.

In addition, there are several numbers of growing research outputs done globally at a fast pace and it is somewhat becoming almost impossible to remain up-to-date with everything that is being published about a particle subject matter at a glance (Briner and Denyer, 2012). Evaluating research documents on citrus waste as a potential animal feed resource for livestock is vital with such studies being carried out with the use of scientometric indicators that will further help to recognize hot, current, and trending research topics, country contribution, international collaboration, and research trends on the subject matter. Therefore, the present study was carried out to assess and analyze research outputs on citrus waste as feed for livestock production.

The procedure often adopted to assess and analyze scientific work done on a particular subject matter is referred to as scientometrics or bibliometrics, which is different from systematic evaluation and literature reviews. The foremost objective of the scientometric analysis is to analyze trends of research, main research themes, top-cited articles in the field, global, national, and local impact, scientific contributions, and vital actors in a particular field. Several top-notch scientific publications including the reports of Khatun and Ahmed (2011), Loomes and van Zanten (2013), and Ekundayo and Okoh (2018), have been carried out by utilizing the scientometric tools to evaluate precise research done in different fields of studies all over the world.

The present study was supported by a scientometric analysis sustained on the data existing in the databases traversed for this research. Additional statistical, data mapping methods, mathematical calculations, and procedures were also added to boost the results of this research work. Therefore, the key drive of this study was to assess the global trend of research outputs on livestock production advanced by the utilization of plant-based alternative resource (citrus waste), by analytically discussing the scholarly position in the production of livestock production using citrus waste, which presently is a growing interest globally and they are the two interconnecting areas of the present study.

Conversely, the present study reported several high-flying categories on the utilization of citrus waste for livestock production studies, for instance, authors, research outputs, distribution of countries, the global trends of citation, thematic evolution of authors' keywords, words, keywords, and trending topics on the subject matter. The outcomes from this study have a robust prospect of increasing the disciplinary knowledge bank of citrus waste as an alternative feed resource for livestock production. By exploring outputs on scholarly high-flying work, this study will help in recognizing areas of potential research gaps and research dynamics on citrus waste and livestock production globally. Furthermore, we are optimistic that this study will be valuable to research experts, livestock industry, and other stakeholders in this field. The study will also help to advance and explore scientific studies done in livestock production and propose probable future research prospects.

## Materials and Methods

### Definitions of Analyzed Data Terms

The analysis package (Bibliometrix) that was used for this study is a suitable tool known for precise/accurate package employed for processing data from articles/publications including file conversion, duplicate matching and merging, similarity normalization for network analysis, descriptive analysis term extraction, and matrix building (Aria and Cuccurullo, 2017). The data matrices used were built from research article datasets, such as words, authors, countries, keywords, and references for outputs such as cocitation, coupling, collaboration, multiple correspondences, and conceptual framework analyses. The bibliometric graphical coupling happens between two (2) research articles of say “a and b” that had their reference lists mentioned/cited from at least one common source (Ekundayo and Okoh, 2018). The aggregate number of bibliographical coupling that ensues between research articles “a and b” or co-authorship in networking and scientific collaboration denotes the strength of the network (Aria and Cuccurullo, 2017). A specific network represents relationships in a classification/system as a group of nodes and links (Zhang et al., 2012). Meanwhile, the conceptual framework assessment uses the K-means grouping/clustering and other measurement techniques to categorize clusters/groups of shared concepts recognized in bibliographic groupings/collections. This K-means depends on word co-occurrences in a research output/publication dataset (Aria and Cuccurullo, 2017). Furthermore, scientific output or a researcher's contributions/influences in a research niche area (field) is analyzed based on Lotka's law. This law can be said to be an inverse square law, which defines how often researchers produce articles in their niche area/field (Lotka, 1926).

### Retrieval of Data Used for Analysis

The present study made use of published scientific documents on citrus waste linked with livestock production research outputs, and they were obtained from the combination of Scopus and Web of Science (WOS) archive on 15 July 2021. Scopus and WOS databases hosted reliable and efficient high-impact scientific publications (Mansoori, 2018; Repiso et al., 2018; Orimoloye et al., 2020). Hence, the current study made use of Scopus and WOS to gather data for the proposed objective. The advanced search function in Scopus and WOS was used because both databases allow for building long and composite search queries. Ordinarily, in research publications that involve scientometric review, one database can be utilized because scientometric indices and literature mapping are hard to perform on articles retrieved from more than one different database (Sweileh, 2020a,b). Conversely, it has been reported that using only one database for information retrieval may limit the inclusion of some significant articles that may be needed for analysis on a particular subject matter (Mansoori, 2018; Orimoloye et al., 2020). The use of Scopus and WOS databases will guarantee 100% inclusion of PubMed. Therefore, Scopus and WOS are guaranteed to have an all-inclusive collection of articles in PubMed and other scientific databases.

### Search Strategy Used for Data Collection

For us to successfully create a search query that is appropriate to collect most of the associated volume of research publications with the slightest false-positive outcome, we embarked on an exhaustive literature review research on the subject matter/topic, and principally on studies and systematic reviews to acquaint ourselves with most of the likely keywords related to the search topic. This method of search strategy for data collection has also been used by several other authors in other research fields (Milan et al., 2013; Mukhopadhyay, 2014; King et al., 2018; Fesseha et al., 2020). The modest technique that we adopted in the search for data collection was to use the title/abstract search methodology for keywords related to “citrus waste” and “livestock.” Howbeit, adopting such a strategy may lead to the retrieval of a huge number of documents that may not be useful for our study. Therefore, to streamline the title/abstract method that was used, a particular constraint was employed that included the presence of certain “terms” related to citrus waste or livestock production in addition to the title/abstract strategy.

### Search Query Employed for Data Collection for This Study

The search query used for the present study comprised of specific phrases related to citrus waste with precise phrases related to livestock production which were entered into the title/abstract search engine. This was followed by some specific terms as a restriction to lessen and remove irrelevant research publications that are not part of the objective of the study. The search queries that we used for WOS and Scopus are given below.

### 1. Scopus

578 document results(TITLE (citrus^*^ AND (waste^*^ OR feed^*^ OR peel^*^ OR pulp))) AND (ruminant^*^ OR livestock OR poultry OR sheep OR goat^*^ OR rumen OR broiler^*^ OR pig^*^ OR swine OR turkey) AND (LIMIT-TO (DOCTYPE, “ar”)).

### 2. Web of Science

187 results from Web of Science Core Collection for:citrus^*^ AND (waste^*^ OR feed^*^ OR peel^*^ OR pulp) (Title)Refined By: Search within all fields: Ruminant^*^ Or Livestock Or Poultry Or Sheep Or Goat^*^ Or Rumen Or Broiler^*^ Or Pig^*^ Or Swine Or Turkey

Document Types: Articles Clear all.

### Analysis and Processing of Data

**Figure 1:**
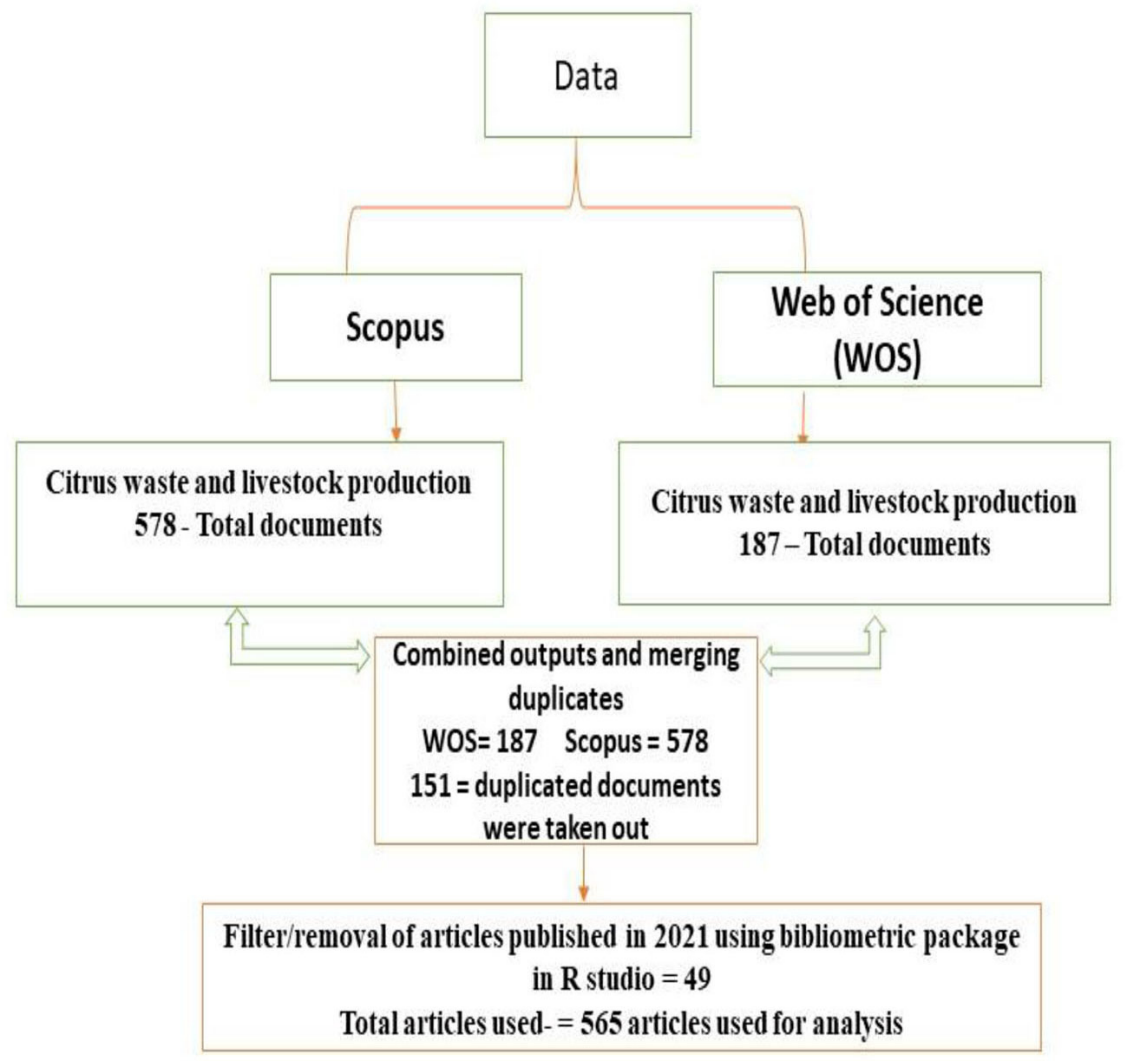
Diagrammatic representation showing the inclusion and exclusion criteria for out-puts selection.

The current study analyzed all collected data from Scopus and WOS databases by using RStudio v. 4.0.4 software with bibliometrix R-package for scientometric elements (Aria and Cuccurullo, 2017). All retrieved data were then imported into R Studio and refined into a bibliographic data form before they were structured to remove duplications that may arise from the two databases (Scopus and WOS) that were used (Ekundayo and Okoh, 2018). Before processing the data, the removal of stop words that are not relevant to the current study was also carried out. Likewise, similar concepts of terms were normalized before analyzing the data. A graphic representation of the retrieval and analysis of data is given in Figure 1. To avoid article duplication extracted from the two databases, all replicated peer-reviewed articles were restricted to one record in the analysis. Furthermore, for visualization, the names of authors, author's keywords (DE), and keywords plus (ID) were extracted. All retrieved data were reviewed for variant names, spelling errors, and associations. For keywords (DE) and keywords-plus (ID), the subject of the present study (citrus waste and animal feed) was given a primary term to terms. Furthermore, the co-occurrence of a phrase in the keywords (DE set) and keywords-plus (ID set) of authors in the dataset was assessed as a group made of the two sets (DE and ID) that converge.

In addition, we employed necessary commands/functions of bibliometrix R-package to analyzed retrieved data for the following descriptive result, citation assessment, authors' scientific performance/productivity. Bibliometric scientific collaboration and networks (e.g., citation, author keyword, and keywords-plus, author, nation links) and bibliometric graphical coupling (keyword co-occurrences and co-citation) were calculated and visualized from bibliometric bipartite (two-way) collaborations/networks of rectangular yardsticks/matrices of research articles × attributes/traits. For instance, the formula for a characteristic bibliometric connection/network is given as Network (*N*) = *X* × *N*^*T*^;

Where *X* is a bipartite/biclustering (two-way) network matrix consisting of research articles × attribute/traits (e.g., nations, authors, citations, and keywords).

*N* is a dimensional/symmetrical matrix *N* = *N*^*T*^.

Likewise, a graphical model was created for all collaborations/networks by utilizing the force-directed algorithms (Fruchterman) applied in the networkPlot command/function of the bibliometrix R-package. Consequently, all the collaborations/networks were normalized/standardized by utilizing Salton's cosine coefficient, proximity indices (association strength), Simpson's coefficient (inclusion indices), and Jaccard's similarity indices among nodes of a network (Aria and Cuccurullo, 2017). In addition, the k-means groupings/clustering were performed on keywords to evaluate concepts in citrus waste as livestock food research field by utilizing the function of a conceptual framework of the bibliometrix R-package software. This function employed Porter's restricting/stemming algorithm (Porter, 1980) to moderately adjusted words to their exact/root form.

## Results

**Figure 2:**
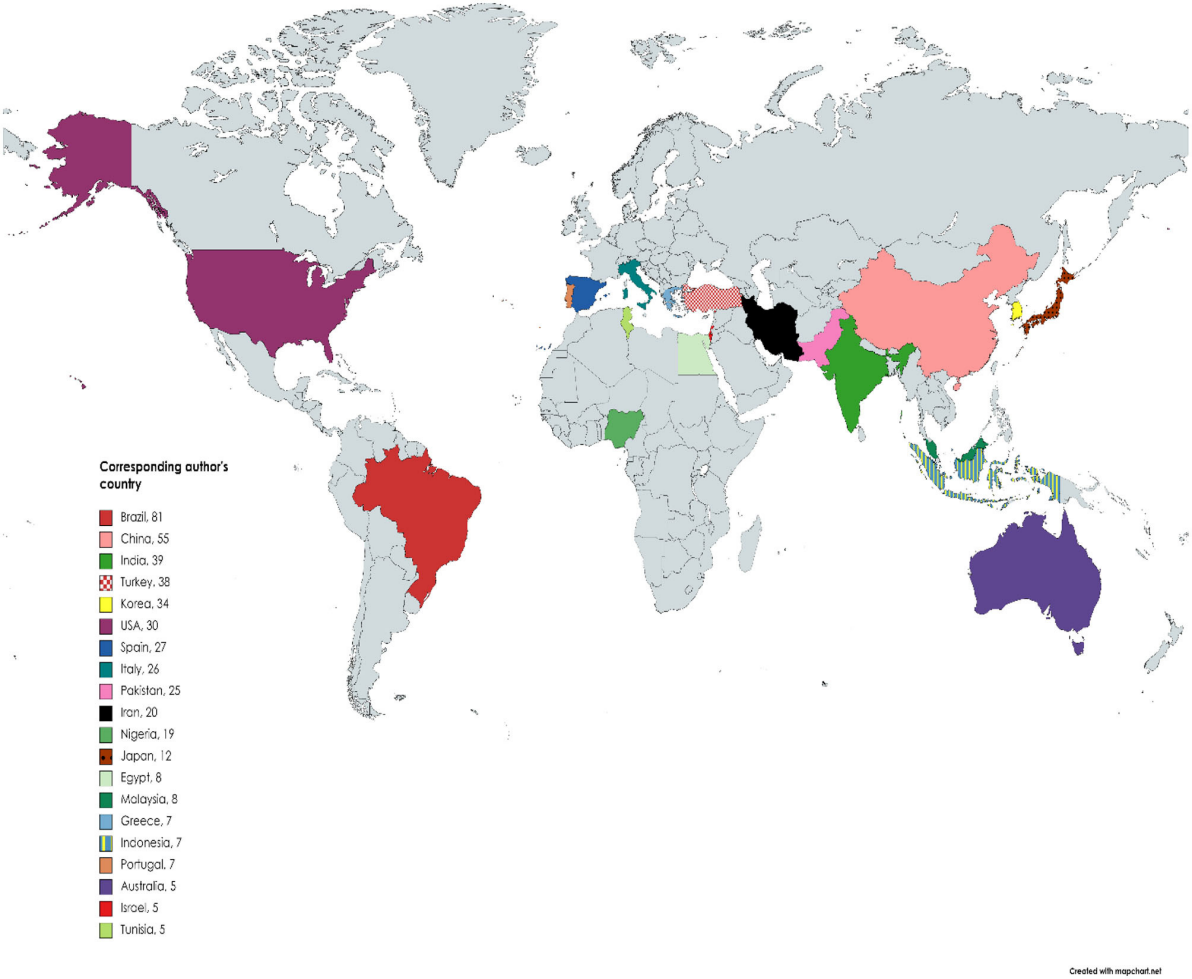
Spatial mapping of the top 20 most productive nations based on number of research articles on citrus waste and animal feed studies (Corresponding author's countries). Gray color areas depict the zones that are not among the top 20 nations.

**Figure 3:**
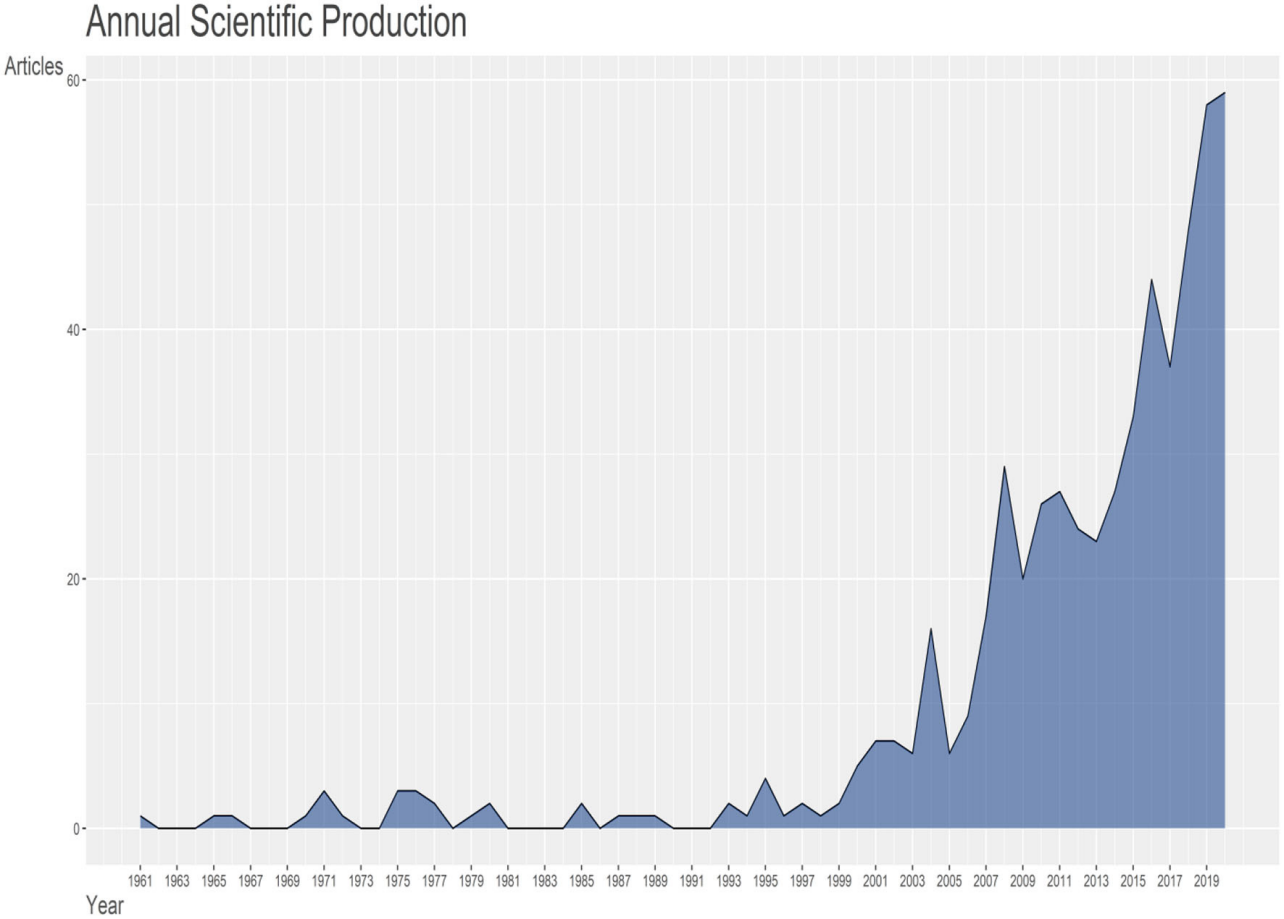
Trends of annual scientific publications (from 1961 to 2020) in citrus waste and animal feed research with an annual growth rate of 10.20%. Citrus waste and animal feed research studies showed several fluctuations (downward and upward trends) in research outputs between 1961 and 2005.

From our results, 565 articles were published between the year 1961 to 2020 of the study period; the analysis features are presented in Table 1. The outputs for the surveyed periods comprise 2,069 authors, with 8 single author, 0.273 articles per author (3.66 authors per article), a collaboration index of 3.71 and 4.88 co-authors per article. With the exemption of eight authors who were single authors, all other authors (2,061) had multi-author research outputs. An average of 17.26 citations per article was documented during the study period. Likewise, Figure 2 shows publications of global mappings associated with citrus waste and animal feed research for livestock production for the top 20 most active nations (based on corresponding authors' countries). Among the five most productive nations, Brazil ranked first in aggregate numbers of articles (*n* = 81), followed by China (*n* = 55), India (*n* = 39), Turkey (*n* = 38), and Korea (*n*= 34), respectively, among other nations. In Figure 3, the present result showed a trend in research outputs on citrus waste and animal feed for livestock production with an annual growth rate of 10.20% (Figure 3). From the values in Figure 3, it can be seen that there were several fluctuations (downward and upward trends) in research outputs between 1961 and 2005 during the studied period; however, there appears to be a steady growth after 2005 till date (Figure 3). The peak year of article publication in citrus waste and animal feed production research field was in the year 2020 with a total output of 59 articles (Figure 3). The average article citations (AAC) of most cited countries in the field of citrus waste and animal feed research for livestock feed showed that Mauritius (*n* =179), United Kingdom (*n* = 118), Israel (*n* = 52.80), and Germany (*n* = 50.66) lead the chart, respectively (Table 2). Publication outputs related to citrus waste and animal feed research for livestock production for the top 20 most active countries are shown in Table 3. Brazil is rated first in terms of the aggregate number of articles published (*n* = 81), the next country was China (*n* = 55), followed by India (*n* = 39) and Turkey (*n* = 38) among others, respectively. The frequency of research outputs varied among the top 20 nations from 0.00951 to 0.15399. Furthermore, the nations that topped the chart with multiple country publications and networking include Italy which is placed in the first position (*n* = 5), Brazil, Korea, and Iran which are tied in the second position (*n* =3), then followed by China and Egypt (*n* = 2), which are tied in the third position, respectively. Conversely, the nations ranked in top positions for single country publication of research outputs in the field of citrus waste and animal feed research for livestock production are Brazil (*n* = 78), China (*n* = 53), India (*n* = 38), Turkey (*n* = 37), and Korea (*n* = 31) among others (Table 3).

**Table 1:** General information of retrieved published documents on citrus waste as animal feed for livestock production from Scopus and WOS data bases.

**Main information about data**	
Timespan	1961:2020
Sources (Journals, Books, etc)	303
Documents	565
Average years from publication	9.34
Average citations per documents	17.26
Average citations per year per doc	2.105
References	13,939
**Document types**	
Article	561
Article; proceedings paper	4
**Document contents**	
Keywords plus (ID)	3,592
Author's keywords (DE)	1,632
**Authors**	
Authors	2,069
Author appearances	2,759
Authors of single-authored documents	8
Authors of multi-authored documents	2,061
**Authors collaboration**	
Single-authored documents	9
Documents per author	0.273
Authors per document	3.66
Co-authors per documents	4.88
Collaboration index	3.71

**Table 2:** The top 20 most cited countries in terms of average article citations (AAC) in the field of citrus waste as animal feed for livestock production from 1961 to 2020.

**S/N**	**Country**	**Total citations**	**Average article citations**
1	China	1,338	24.32
2	Brazil	733	9.04
3	Spain	650	24.07
4	India	639	16.38
5	Italy	542	20.84
6	Pakistan	542	21.68
7	Turkey	534	14.05
8	USA	506	16.86
9	United Kingdom	472	118
10	Korea	424	12.47
11	Malaysia	267	33.37
12	Nigeria	267	14.05
13	Israel	264	52.80
14	Portugal	225	32.14
15	Japan	194	16.16
16	Mauritius	179	179
17	Germany	152	50.66
18	Iran	146	7.30
19	Greece	142	20.28
20	Algeria	104	34.66

**Table 3:** The top 20 publications by countries in the field of citrus waste and animal feed production for livestock research.

**S/N**	**Country**	**Articles**	**Rank**	**Frequency**	**SCP**	**MCP**	**MCP_Ratio**
1	Brazil	81	1	0.15399	78	3	0.037
2	China	55	2	0.10456	53	2	0.036
3	India	39	3	0.07414	38	1	0.025
4	Turkey	38	4	0.07224	37	1	0.026
5	Korea	34	5	0.06464	31	3	0.088
6	USA	30	6	0.05703	29	1	0.033
7	Spain	27	7	0.05133	27	0	0
8	Italy	26	8	0.04943	21	5	0.192
9	Pakistan	25	9	0.04753	24	1	0.040
10	Iran	20	10	0.03802	17	3	0.150
11	Nigeria	19	11	0.03612	19	0	0
12	Japan	12	12	0.02281	12	0	0
13	Egypt	8	13	0.01521	6	2	0.250
14	Malaysia	8	13	0.01521	8	0	0
15	Greece	7	14	0.01331	7	0	0
16	Indonesia	7	14	0.01331	7	0	0
17	Portugal	7	14	0.01331	7	0	0
18	Australia	5	15	0.00951	5	0	0
19	Israel	5	15	0.00951	5	0	0
20	Tunisia	5	15	0.00951	4	1	0.200

*SCP, single country publications; MCP, multiple country publications*.

Among the most relevant author keywords in the field of citrus waste and animal feed, citrus pulp (*n* = 48) was ranked first, followed by citrus (*n* = 32), performance (*n* = 23), and pectin (*n* = 22), among other keywords used by authors (Table 4). Furthermore, in Table 5, the top 20 most relevant/productive authors in the field of citrus waste and animal feed were reported. The author A, Pires was placed in the first position (*n* = 14) based on the number of articles; while I, Susin was ranked in the second position (*n* = 13), meanwhile G, Rodrigues was placed in the third position. Two authors, namely, R, Gentil and U, Ruiz, however, maintained the fourth position with 10 article publications each. Based on total citations, the H_index was 6 (TC = 140) for A, Pires who maintained the first position. However, author I, Susin was in the second position with H_index of 6 (TC = 151). The third position was maintained by G, Rodrigues with H_index of 4 (TC = 142).

**Table 4:** Most relevant words used by researchers in the field of citrus waste and animal feed production.

**S/N**	**Author keywords (DE)**	**Occurrence**	**Keywords-plus (ID)**	**Occurrence**
1	Citrus pulp	48	Citrus	290
2	Citrus	32	Fruit	104
3	Performance	23	Plant extracts	83
4	Pectin	22	Antioxidants	77
5	Antioxidant activity	20	Animals	74
6	Antioxidants	18	Citrus fruits	68
7	*Citrus sinensis*	18	Flavonoids	68
8	Sheep	17	Chemistry	57
9	Limonene	16	Metabolism	49
10	Citrus peel	14	Male	48
11	Silage	14	*Citrus sinensis*	41
12	Broiler	13	Unclassified drug	41
13	Citrus peels	13	Fermentation	37
14	Digestibility	13	*Zea mays*	34
15	Essential oil	13	Essential oil	31
16	Adsorption	11	Sweet orange	31
17	Fatty acids	11	Animalia	27
18	Growth performance	11	Adsorption	26
19	Response surface methodology	11	Cattle	26
20	By-product	10	Female	25

**Table 5:** Top 20 relevant/productive authors on citrus waste and animal feed for livestock production.

**S/N**	**Author**	**Rank**	**H_index**	**G_index**	**M_index**	**TC**	**NP**	**% of 565**	**PY_start**
1	Pires A	1	6	11	0.30	140	14	2.47	2002
2	Susin I	2	6	12	0.42	151	13	2.30	2008
3	Rodrigues G	3	6	11	0.42	142	11	1.94	2008
4	Gentil R	4	4	8	0.33	71	10	1.76	2010
5	Ruiz U	4	4	7	0.30	57	10	1.76	2009
6	Zhang Y	5	6	9	0.42	388	9	1.59	2008
7	Qotbi A	5	5	9	0.50	117	9	1.59	2012
8	Seidavi A	5	5	8	0.50	68	9	1.59	2012
9	Thomaz M	5	4	6	0.30	47	9	1.59	2009
10	Watanabe P	5	4	6	0.30	43	9	1.59	2009
11	Ferreira E	6	4	6	0.36	43	8	1.41	2011
12	Pascoal I	6	3	6	0.23	40	8	1.41	2009
13	Ebrahimi A	7	5	7	0.55	64	7	1.23	2013
14	Mendes C	7	5	7	0.35	130	7	1.23	2008
15	Pereira M	7	4	7	0.28	67	7	1.23	2008
16	Tufarelli V	7	5	7	0.55	105	7	1.23	2013
17	Deng X	8	5	6	0.27	161	6	1.06	2004
18	Kim J	8	4	6	0.28	41	6	1.06	2008
19	Lee M	8	3	4	0.16	22	6	1.06	2004
20	Lee S	8	4	6	0.33	42	6	1.06	2010

*NB: % of 565 = total sum of articles published on citrus waste as animal feed for livestock production between 1961 and 2020. NP, number of publications; PY_start, publication year start. Ranking is based on number of publication (NP)*.

**Figure 4:**
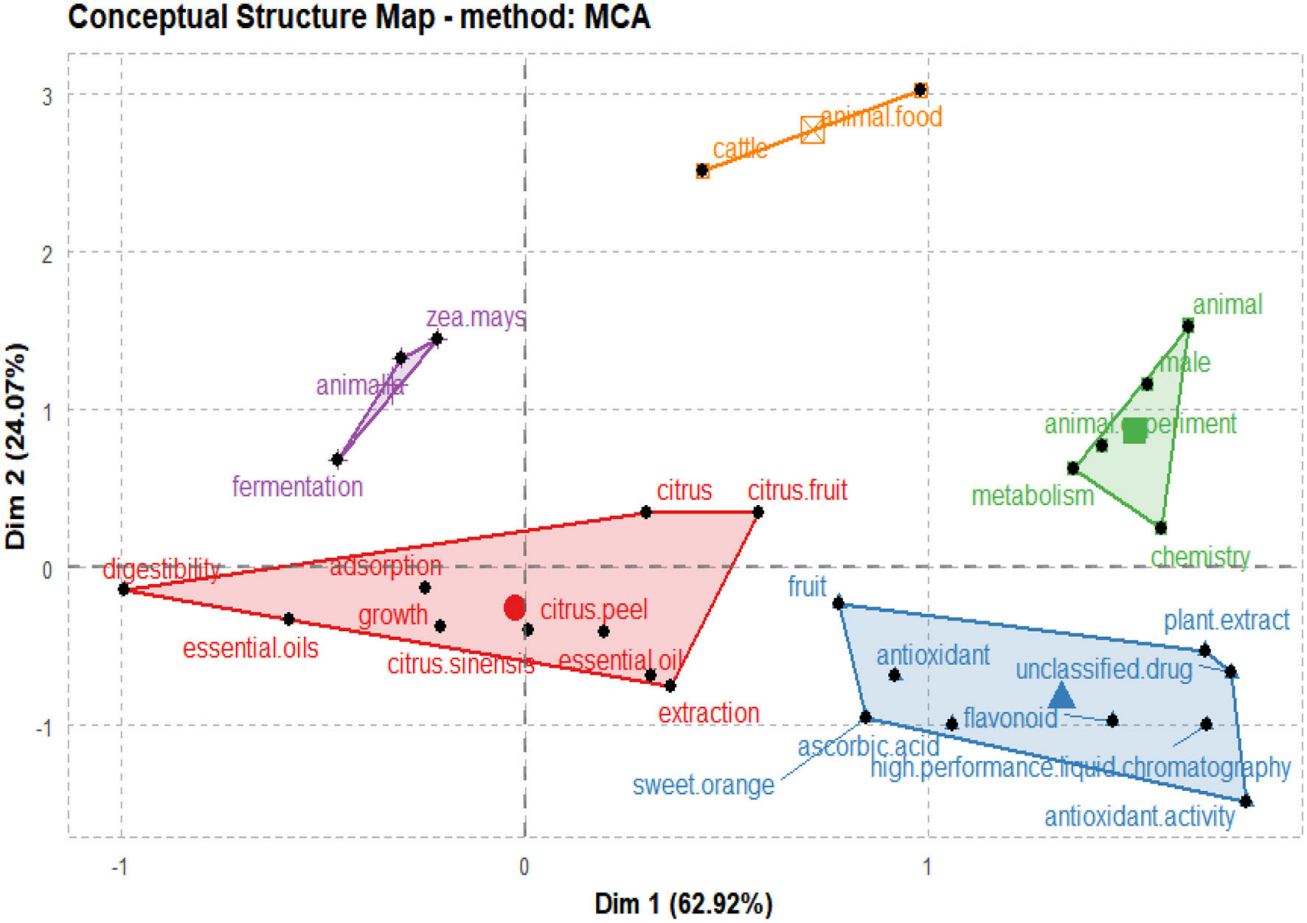
Shared conceptual structural map associated with citrus waste and livestock feed research studies (1961–2020).

The shared conceptual frame maps of retrieved publications of citrus waste between 1961 and 2020 by factorial multiple correspondence revealed five clusters of sizes 10, 9, 5, 3, and 2 with research component focusing on citrus waste as animal feed, for livestock (e.g., cattle), to improve livestock performance (growth and digestibility), as feed supplements to maize (*Zea mays*), as alternative feedstuffs (citrus fruits, peels, and others) and as medicinal plants (antioxidant activities, flavonoid) for improving livestock performance and metabolism (Figure 4). The clusters explained the type of livestock animal commonly fed with citrus waste (cattle and other livestock, including sheep, goat, and chicken), and how citrus waste can be used as feed, that is, as a supplement to maize meal (*Z. mays*) or as a processed feed meal (citrus fruit or citrus peels). The orange cluster showed citrus waste as food for cattle. The purple cluster showed the use of citrus waste as a supplement with maize while the red cluster indicated the parts (citrus fruits, citrus peels, and others) of citrus waste that can be used for livestock feed to improve digestibility and growth of livestock. The blue cluster showed the utilization of citrus waste as medicinal plants (antioxidant), whereas the green cluster revealed the potential of citrus waste for improving animal metabolism (Figure 4).

Table 6 shows the top 20 most cited articles on citrus waste and animal feed based on total citations from 1961 to 2020. The article written by Xu et al. (2007) was ranked in the first position with a total of 280 citations. The article placed in the second position was authored by Mandalari (2007) with a total of 230 citations. The third and fourth positions had D, Ramful (TC = 199) and B. H. Hameed (TC = 175), respectively (Table 6).

**Table 6:** Top 20 most cited articles on citrus waste and animal feed research from 1961 to 2020.

**S/N**	**References**	**Journal name**	**Article title**	**Total citations**	**TC per year**	**Normalized TC**
1	Xu et al. (2007)	Journal of Agricultural and Food Chemistry	Effect of heat treatment on the phenolic compounds and antioxidant capacity of citrus peel extract	280	18.66	6.08
2	Mandalari et al. (2007)	Journal of Applied Microbiology	Antimicrobial activity of flavonoids extracted from bergamot (*Citrus bergamia* Risso) peel, a byproduct of the essential oil industry	230	15.33	5
3	Ramful et al. (2011)	Food Research International	Polyphenol composition, vitamin C content and antioxidant capacity of Mauritian citrus fruit pulps	179	16.27	5.78
4	Hameed et al. (2008)	Colloids and Surfaces A: Physicochemical and Engineering Aspects	Sorption of basic dye from aqueous solution by pomelo (*Citrus grandis*) peel in a batch system	175	12.50	5.98
5	Kyriazakis and Emmans (1995)	British Journal of Nutrition	The voluntary feed intake of pigs given feeds based on wheat bran, dried citrus pulp and grass meal, in relation to measurements of feed bulk	157	5.81	1.83
6	Ashgar and Bhati (2012)	Ecological Engineering	Evaluation of thermodynamics and effect of chemical treatments on sorption potential of Citrus waste biomass for removal of anionic dyes from aqueous solutions	154	15.40	5.73
7	Trebitsh et al. (1993)	Proceedings of the National Academy of Sciences of the United States of America	Ethylene induces *de novo* synthesis of chlorophyllase, a chlorophyll degrading enzyme, in Citrus fruit peel	153	5.27	1.83
8	Shakoor and Nasar (2016)	Journal of the Taiwan Institute of Chemical Engineers	Removal of methylene blue dye from artificially contaminated water using citrus limetta peel waste as a very low cost adsorbent	138	23	8.67
9	Dutta et al. (2011)	Desalination	Application of response surface methodology for preparation of low-cost adsorbent from citrus fruit peel and for removal of methylene blue	131	11.90	4.23
10	Qiao et al. (2008)	Molecules	Characterization of aroma active compounds in fruit juice and peel oil of jinchen sweet orange fruit [*Citrus sinensis* (L.) Osbeck] by GC-MS and GC-O	127	9.07	4.34
11	Malisch (2000)	Chemosphere	Increase of the PCDD/F-contamination of milk, butter, and meat samples by use of contaminated citrus pulp	122	5.54	2.16
12	Sunvold et al. (1995)	Journal of Animal Science	*In vitro* fermentation of cellulose, beet pulp, citrus pulp, and citrus pectin using fecal inoculum from cats, dogs, horses, humans, and pigs and ruminal fluid from cattle	114	4.22	1.33
13	Xu et al. (2008)	Journal of Food Sciences	Minerals, phenolic compounds, and antioxidant capacity of citrus peel extract by hot water	109	7.78	3.72
14	Kamal et al. (2011)	International Food Research Journal	Yield and chemical composition of Citrus essential oils as affected by drying pretreatment of peels	105	9.54	3.39
15	Fagbohungbe et al. (2016)	Bioresource Technology	Impact of biochar on the anaerobic digestion of citrus peel waste	97	16.16	6.09
16	Lagha-Benamrouchea and Madani (2013)	Industrial Crops and Products	Phenolic contents and antioxidant activity of orange varieties (*Citrus sinensis* L. and *Citrus aurantium* L.) cultivated in Algeria: peels and leaves	95	10.55	4.30
17	Schiewer and Patil (2008)	Journal of Hazardous Materials	Modeling the effect of pH on biosorption of heavy metals by citrus peels	92	6.57	3.14
18	Acar et al. (2015)	Aquaculture	Evaluation of the effects of essential oil extracted from sweet orange peel (*Citrus sinensis*) on growth rate of tilapia (*Oreochromis mossambicus*) and possible disease resistance against Streptococcus iniae	91	13	5.06
19	Garcia-Castello et al. (2015)	Lebensmittel-Wissenschaft and Technologie	Optimization of conventional and ultrasound assisted extraction of flavonoids from grapefruit (*Citrus paradisi* L.) solid wastes	88	12.57	4.89
20	Wu et al. (2014)	Journal of Experimental Botany	An integrative analysis of the transcriptome and proteome of the pulp of a spontaneous late-ripening sweet orange mutant and its wild type improves our understanding of fruit ripening in citrus	86	10.75	5

The top 20 journals with the most published articles in the field of citrus waste and animal feed research for livestock production are listed in Table 7. These journals cover a range of fields, including citrus as medicinal plants (antioxidant activities, essential oils, plant extracts, liquid chromatography) chemistry, and livestock production (growth, digestibility, high performance), among others. Journals such as *Revista Brasileira de Zootecnia, Arquivo Brasileiro de Medicina Veterinaria E Zootecnia, Journal of Agricultural and Food Chemistry, Revista Brasileira de Zootecnia* [Brazilian Journal of Animal Science]*, Journal of Animal Science Journal of Dairy Science* reflect active areas in citrus waste and animal feed research. However, the journal *Revista Brasileira de Zootecnia* ranked first (number of publications = 23; H_index = 10) among the journals with the most published articles. This was followed by *Arquivo Brasileiro de Medicina Veterinaria E Zootecnia* (number of publications = 15; H_index = 4). The *Journal of Agricultural and Food Chemistry* and *Revista Brasileira de Zootecnia* [Brazilian Journal of Animal Science] were ranked in the third position with the number of publications being 11 each and H_index being 9 and 7, respectively. Conversely, the *Journal of Animal Science* was ranked in the fourth position among the journals with the most published articles (Table 7).

**Table 7:** The top 25 journals that are relevant in the field of citrus waste and animal feed research from 1961 to 2020.

**S/N**	**Source**	**H_index**	**G_index**	**M_index**	**TC**	**NP**	**PY_start**
1	Revista Brasileira de Zootecnia	10	15	0.434782609	266	23	1999
2	Arquivo Brasileiro de Medicina Veterinaria E Zootecnia	4	6	0.2	59	15	2002
3	Journal of agricultural and food chemistry	9	11	0.160714286	557	11	1966
4	Revista Brasileira de Zootecnia-Brazilian journal of animal science	7	11	0.368421053	122	11	2003
5	Journal of animal science	8	10	0.156862745	255	10	1971
6	Journal of dairy science	9	10	0.191489362	292	10	1975
7	Asian-Australasian journal of animal sciences	4	7	0.25	67	7	2006
8	Food chemistry	7	7	0.7	208	7	2012
9	Pakistan journal of nutrition	3	6	0.2	39	7	2007
10	Small ruminant research	6	7	0.285714286	193	7	2001
11	Animal feed science and technology	5	6	0.119047619	98	6	1980
12	Asian journal of chemistry	3	6	0.25	36	6	2010
13	Journal of food science	4	6	0.076923077	150	6	1970
14	Journal of food science and technology	4	6	0.285714286	133	6	2008
15	Meat science	6	6	0.333333333	104	6	2004
16	Molecules	6	6	0.428571429	199	6	2008
17	Applied chemistry for engineering	3	4	0.6	17	5	2017
18	Bioresource technology	5	5	0.416666667	209	5	2010
19	Journal of animal and veterinary advances	3	4	0.2	18	5	2007
20	Journal of essential oil research	3	4	0.166666667	16	5	2004
21	Journal of the science of food and agriculture	4	5	0.121212121	159	5	1989
22	Pakistan journal of zoology	4	5	0.363636364	49	5	2011
23	Industrial crops and products	4	4	0.444444444	245	4	2013
24	Journal of food measurement and characterization	2	4	0.666666667	17	4	2019
25	Journal of food processing and preservation	3	4	0.272727273	27	4	2011

*NP, number of publication; TC, total citations; PY_start, publication year sta*.

**Figure 5:**
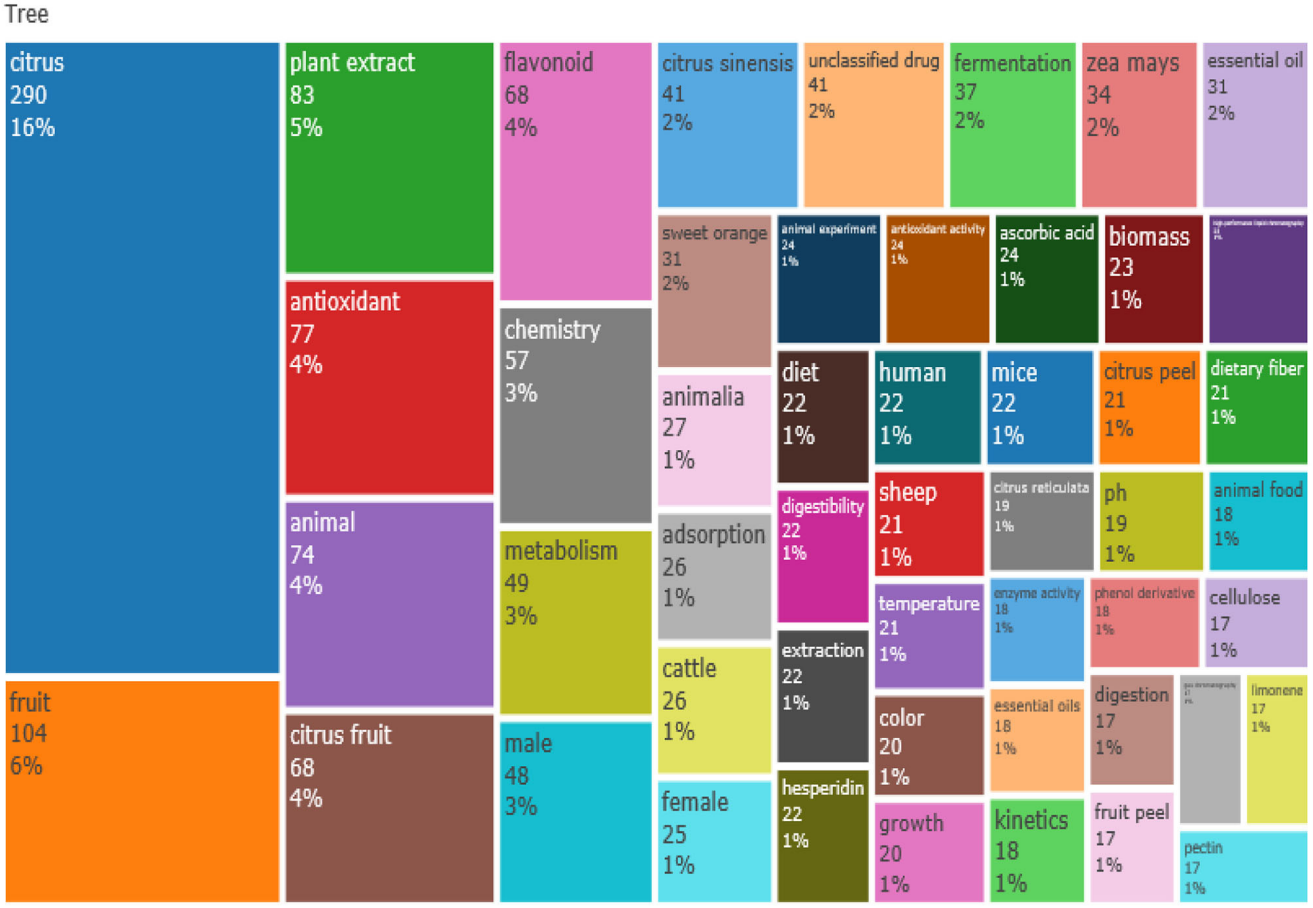
Tree map of discipline distribution in the field citrus waste and livestock feed research.

**Figure 6:**
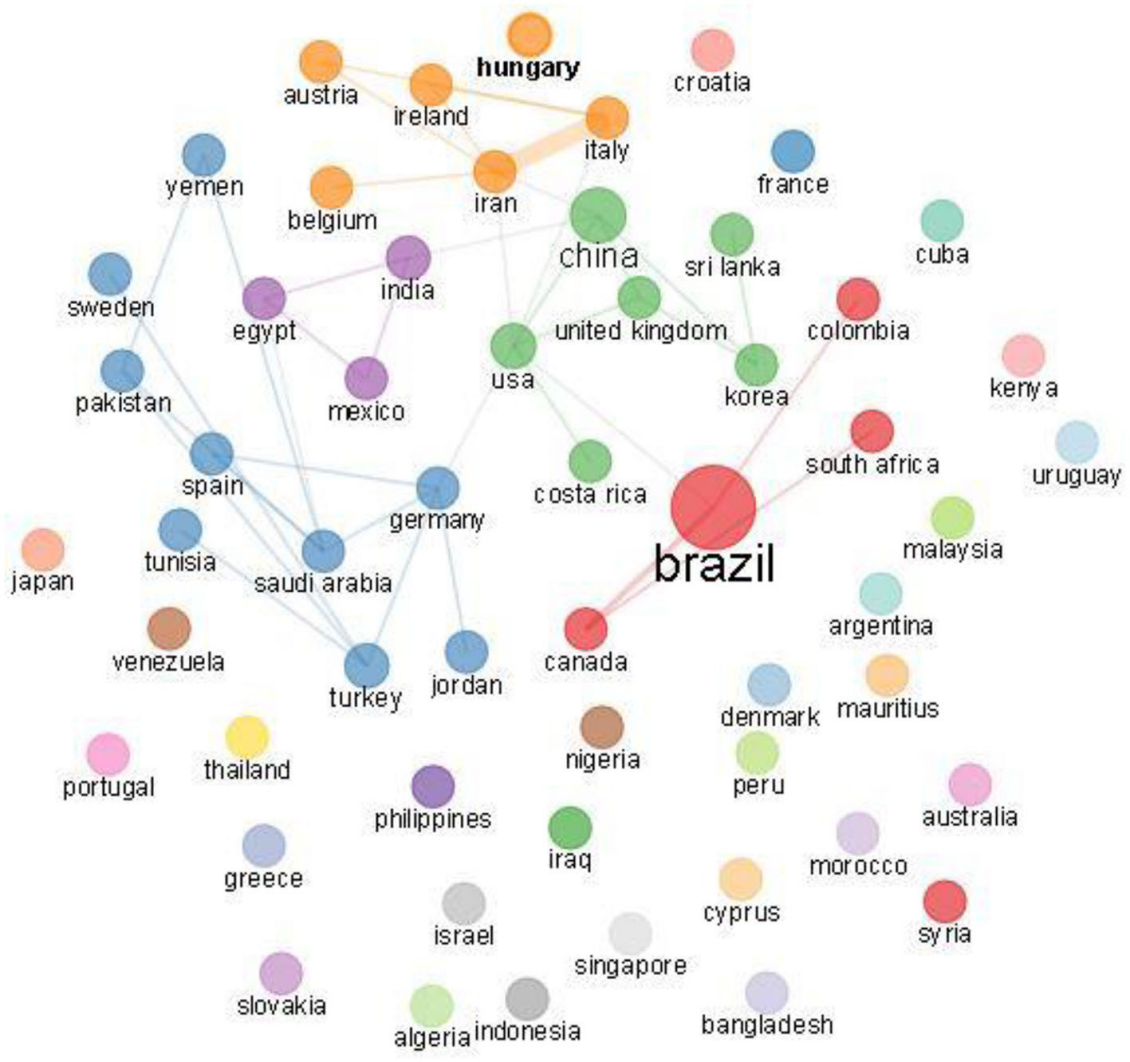
Thirty nations' collaboration networks on research out-puts on citrus waste and animal feed from 1961 to 2020.

From statistical assessment of the articles related to the use of citrus waste for animal feed production revealed that we can deduce that animal feed produced from citrus waste for livestock farming involves several research directions, including Animal Husbandry (such as cattle and sheep farming), Chemistry (antioxidants, adsorption, extraction), and Medicinal Botany (extracts, animal experiment), among others. The current study presents a better motivation for the advancement and a large space for research in the field of citrus waste recovery and animal feed (Figure 5). Furthermore, Figure 6 shows a network visualization map of nations' collaboration, depicting 30 countries that are involved with citrus waste as animal feed. Each node in the network is an individual nation and the diameter of the node corresponds to the number of publications by each nation. The strokes/lines denote the paths of networking between nations and the thickness of strokes/lines signifies the degree of collaboration between the nations. Collaboration links ranged from 1 to 20. Italy had a high number of collaborations (*n* = 20), followed by the United States (*n* = 19), and Iran (*n* = 13). However, collaboration links for developing countries such as Egypt, Nigeria, and Indonesia had very few links/networks compared to those from developed countries.

The top 10 most prolific institutions with at least 10 publications are listed in Table 8. The Islamic Azad University in Iran (number of articles = 31) was ranked first; University of Agriculture in Pakistan (number of articles = 23) was ranked second, and the University of São Paulo in Brazil (number of articles = 15) was ranked the third position, respectively, among others. Conversely, from the 10 most prolific institutions that were ranked, two of the institutions (University of São Paulo and Universidade De São Paulo) were from the same country, that is, Brazil (third and eighth position), while another country, Turkey had two institutions (Istanbul University and Canakkale Onsekiz Mart University) in the fifth and eighth positions of most prolific institutions (Table 8).

**Table 8:** The top 10 most productive institutes on citrus waste and animal feed for livestock production with at 10 article publications.

**S/N**	**Affiliations**	**Countries**	**Article number**	**Position**
1	Islamic Azad Univ	Iran	31	1st
2	University of Agriculture	Pakistan	23	2nd
3	Univ São Paulo	Brazil	15	3rd
4	Not reported	N/A	14	4th
5	Istanbul Univ	Turkey	13	5th
6	University of Florida	USA	12	6th
7	Huazhong Agricultural University	China	11	7th
8	Canakkale Onsekiz Mart Univ	Turkey	10	8th
9	Universidade De So Paulo	Brazil	10	8th
10	Zhejiang University	China	10	8th

## Discussion

The current study of citrus waste and animal feed for livestock production examined global research trends between 1961 and 2020 based on data collected from Scopus and WoS. We observed that the number of publications on citrus waste and animal feed for livestock production increased non-linearly from one article in 1961 to 565 articles in 2020. Conversely, there were fluctuations in research outputs on citrus waste and animal feeds from 1961 to 2009. These fluctuations, however, experienced a steady rate of increase which was observed from 2005 (*n* = 6) to 2020 (*n* = 59) resulting in an annual increase of 10.20% of article publications. The increase in research on citrus waste and animal feed suggests that more researchers have now developed more interest in this area in the past 15 years. This trend may likely be a result of the continual exploration of plant-based alternative feed resources to advance the search for cheaper and helpful animal feed for livestock production. Several authors have reported the prospects of using citrus waste (pulp) as animal feed to improve milk yield in cattle (Malisch, 2000), feed intake in pigs (Kyriazakis and Emmans, 1995), the growth rate in fish (Acar et al., 2015), and to promote growth performance, intestinal morphology, humoral immunity, and egg quality in poultry (Nazok et al., 2010; Basir and Toghyani, 2017; Seidavi et al., 2020). Furthermore, from the annual scientific production graph in Figure 3 (with an annual increase of 10.20%), there is a strong indication that more scientific outputs on the use of citrus for animal feed will further increase in the future. Although it seems that the utilization of citrus/citrus waste as animal feed in Agriculture has not been fully exploited, citrus waste (peels or pulp or essential oils) has been used in other fields such as Chemistry, Medicinal plant, and Botany among others (Kamal et al., 2011; Ramful et al., 2011; Lagha-Benamrouchea and Madani, 2013). Its successful application in the field of Botany, Chemistry, and Medicinal plants is an indication of its novel potential for animal feed and livestock production. With the ever-growing challenge of price increase in conventional animal feed and sometimes scarcity in obtaining animal feed for livestock production in some countries, and especially in developing countries, this may further drive the present generation of new scientific knowledge, toward a significant rise in the number of research studies that will be done on this subject matter.

As with other research areas, it was observed in the current study that most of the leading authors driving the use of citrus waste (peel, pulp, leaves) as an alternative animal feed resource for livestock production were mostly from developed countries such as China, Brazil, the United States, Korea, and Turkey, with a few from very low-income countries (such as Nigeria and Egypt), thus follow the associated trend of low productivity of these countries. The foremost countries with collaboration links on citrus waste and animal feed research studies showed a collaboration allied was mostly among researchers from developed and highly financially stable countries, including the United States and Italy. This observation appears to be a similar trend for collaboration networks of countries in many other research fields (Orimoloye and Ololade, 2021; Smith et al., 2021). A common trend in bibliometric studies shows that alliances between developed and developing countries are scarce in several scientific fields (Ekundayo and Okoh, 2018; Orimoloye and Ololade, 2021). Collaboration in scientific research from both intra- and international institutions between developing and developed countries could foster more robust opportunities for pulling resources (funds and facilities) and more manpower for division of labor to tackle important research gaps in the field of citrus waste and animal feed research.

According to Zhang et al. (2010) and Peng et al. (2015), the economic strength/prowess of a certain nation motivates their research priority and productivity. The situation of food insecurity, sustainability, and increase in price of conventional animal feed in most developing countries, and mostly in countries from sub-Saharan Africa should spark up more drive in researchers in these countries to explore the prospect of doing more studies on the use of citrus and/or waste as animal feed for livestock production.

Several parts of citrus waste (including peel and pulp) are known to be high in lingo-cellulosic substances, which are reported to be a rich source of compounds for the production of new and sustainable chemicals and fuels (Ranzi et al., 2008). Furthermore, several scientists have reported the use of citrus waste (valorization techniques) for biofuels through the process of gasification, thermolysis, combustion, and pyrolysis (Miranda et al., 2009; Zapata et al., 2009; Kim et al., 2015; Volpea et al., 2015; Alvarez et al., 2018). More research studies geared toward exploring innovative ways of adopting the use of citrus waste for animal feed can, however, not be over-emphasized. According to Chavan et al. (2018), waste from citrus fruits is a very essential resource material that can be utilized to boost the global food systems both in the area of farming and in the industrial sectors.

In Table 6, it was observed that, some of the most frequently cited articles were studies associated with the utilization of citrus waste (peel, pulp, and others) as an alternative feed resource for improving livestock production such as milk yield, growth performance, immunity booster, and feed intake in different farm animals (cow, pig, fish, poultry) among others. For example, some articles reported the use of citrus parts (such as peels, pulps) as waste to

(i) Increase the PCDD/F-contamination of milk, butter, and meat samples by use of contaminated citrus pulp (article by Malisch, 2000).
(ii) The voluntary feed intake of pigs given feeds based on wheat bran, dried citrus pulp, and grass meal, in relation to measurements of feed bulk (article by Kyriazakis and Emmans, 1995).
(iii) Evaluation of the effects of essential oil extracted from sweet orange peel (*Citrus sinensis*) on the growth rate of tilapia (*Oreochromis mossambicus*), and possible disease resistance against *Streptococcus iniae* (article by Acar et al., 2015) were among other excellent articles that were frequently cited in this research field.

Conversely, it can be observed as seen in Table 6 that, other authors focused on the medicinal potential of citrus waste (peel, leaves, and pulp) as a functional food for animals and as antimicrobial and antioxidant agents (articles by Mandalari et al., 2007; Lagha-Benamrouchea and Madani, 2013). These novel research outputs are a pointer to the many other value addition prospects that can be derived in the use of citrus (pulp, peels, waste, leaves) as an essential alternative plant resource in the farming and food industry. According to Chavan et al. (2018), solid waste obtained from citrus is a good source of rich alternative plant-based sources that can be used as ingredients for livestock feed. In addition, Moreira et al. (2004) reported that as a very cheap and easy way of disposal for most industries and crop farms worldwide, that is, citrus waste is converted (in fresh or dried form) into animal feed for boosting livestock production.

China, Brazil, and India occupied the foremost positions among the top 20 nations that are in active research in the use of citrus waste as animal feed in terms of number of articles and citations among others (Tables 2, 3). One major reason for any country to fall within the category of more number of article production and citations in a given field may be attributed to the nations' economic strength and access to research facilities and funding (Liu et al., 2013; Peng et al., 2015; Zyoud, 2017). In addition, the increased productivity by foremost nations in this field could be attributed to the fact that China, Brazil, and India are known to be the largest producers of citrus fruits globally with a production yield of 44.1, 19.7, and 14.0 (million tons) of the plant (Knoema, 2019). However, although, the United States has always shown dominance in most research fields (Bundschuh et al., 2013; Geaney et al., 2015; Bruggmann et al., 2017), in the current study, the United States maintained the sixth position, judging from the list of top 20 most published articles in citrus waste and animal feed research (Table 3). The reason for the drop in position for the United States among the nations that had more article outputs is not known. However, the United States is reported to be ranked as the fifth largest producing country in terms of citrus fruit production (Knoema, 2019). This may be a contributing factor to their ranking in terms of article outputs in citrus waste and animal feed research.

Conversely, there is a relatively low contribution to research outputs on citrus waste and animal feed studies by developing countries including countries from Africa (with only Nigeria, Egypt, and Tunisia making the list in the top 20 countries) as observed from the present study (Table 3). This observation may not be unrelated to the fact that most research works done in these countries are often independent studies or self-funded which do not attract research funding from their government or non-governmental organizations.

There are very few observed situations of countries with multiple collaborations (multiple country publications) on citrus waste and animal feed research with Italy having the highest occurrence of multiple collaborations (*n* = 5) as seen in Table 3. Most countries in the list of top 20 most productive countries do not have collaborations (Table 3). This may not be unconnected to the fact that the present field (citrus waste as animal feed for livestock production) seems new, but it is gradually gaining recognition globally with an annual growth rate of 10.20% (Figure 3). However, from our observation, it is expected that more countries may likely get involved in multiple country collaborations judging from the current annual growth rate of research outputs being produced in recent years in this field. Collaboration in scientific research from both intra- and international institutions between developing and developed countries will bring about more robust opportunities for pulling resources (funds and facilities) and more manpower for division of labor to tackle vital research gaps in the area of citrus waste and animal feed for livestock production research.

Likewise, it was noticed from the current study that there was a swing in the rankings among the top 20 countries who were most active in the field of citrus waste and animal feed research when outputs were assessed using the criteria of total citations (TC) per nation (Figure 2 and Table 2). A similar observation was also reported in another scientometric study (Ekundayo and Okoh, 2018). The explanation for this unexpected swing in rankings when using the total number of citations to judge the publication outputs of a nation or an author may indicate its unreliability as an accurate measure for productivity. The rate of citations does not reflect the publication outputs of a nation or an author (Fricke et al., 2013). This is because, according to Fricke et al. (2013), the smaller the number of articles used for valuation, the greater the impact of a few regularly cited articles. In several cases, some researchers have been noticed to engage in self-citations, while others give inaccurate citations when writing their manuscripts which in turn may produce false qualitative and quantitative metrics of total citations about a particular author or country (Ekundayo and Okoh, 2018).

As envisaged, the present study provided some hints of the recent research hotspots in the field of citrus waste and livestock feed for the study period. A co-word biclustering (two-way) evaluation was done to reveal the scientific research hotspots in the niche area, where there were five clusters representing the hotspots, respectively. From the k-means analysis, the most regularly focused hotspot research and research disciplines (including article outlets) associated with citrus waste and animal feed study revealed the research hotspot during the surveyed period which included citrus waste (including citrus peels, citrus fruits such as sweet orange, *C. sinensis*) utilized to formulate food for improvement of livestock (e.g., cattle, animal food), citrus medicinal potentials (antioxidant activities, essential oils, plant extracts, liquid chromatography, chemistry), and the effect of citrus waste feed on livestock performance (growth, digestibility, high performance), which are commonly linked to citrus waste and animal feed research. The hotspot focus for citrus waste and animal field span through some disciplines such as animal feed nutrition, chemistry, bio-medicinal research, among others (such animal physiology, microbiology, and botany research).

Some topics, such as digestibility, growth, citrus fruits, citrus peels, adsorption, and essential oils have often been interesting for scientists/researchers. Sweet orange, *C. sinesis* and citrus peels and fruits seem to be the parts of “citrus waste” that have been consistently reported to be used for livestock feed. Specifically, research on antioxidants, flavonoids, ascorbic acids, and antioxidant activities has become a hotspot, probably because of the potential of the citrus peel to be used as feed, as well as for medicinal purposes in livestock production (Benavente-Garcia and Castillo, 2008). Meanwhile, metabolism and chemistry seem to be new foci among the research field of citrus waste and livestock feed.

In addition, the keywords and research discipline observed from our study showed that some of the efforts made by authors to advance research work on the application of citrus waste as an alternative plant-based feed resource for livestock production were to gain an understanding of the prospect of the practical approach involved in animal feed/nutrition strategies for livestock farming. These findings (most regularly revealed keywords) were supported by other conceptual framework indicators such as the treemap and the keywords network visualization diagram (Figure 5). More important to note, is that, in the study by Acar et al. (2015), whose article happens to be among the top 20 most cited articles, their findings reported on the innovativeness of using essential oil extracted from sweet orange peel (*C. sinensis*) on the growth rate of tilapia fish when compared to the basal feed. Likewise, Acar et al. (2015) reported the potential of using the essential oil extracts from orange peel to resist disease infection against. *iniae*, a common microbial pathogen of the fish (Table 6). The novel study by Acar et al. (2015) is an essential aspect (among other novel research works) that revealed the innovative use of citrus waste as both a useful feed ingredient for livestock and a potential medicinal plant agent that can be used to mitigate against harmful microorganisms.

The use of citrus waste as animal feed to improve milk yield in cattle (Malisch, 2000), feed intake in pigs (Kyriazakis and Emmans, 1995), the growth rate in fish (Acar et al., 2015), and growth performance, intestinal morphology, humoral immunity, and egg quality in poultry (Nazok et al., 2010; Abbasi et al., 2015; Basir and Toghyani, 2017; Seidavi et al., 2020) have been reported. However, we noticed from the retrieved data in our study that, most of the work done on citrus waste as animal feed for livestock production and its research directions (seen from keywords network visualization, treemap, and conceptual structure map) were carried out on broilers, swine, sheep, goat, cattle, and fish. These studies mostly reported the use of citrus pulp (as waste) as feed ingredients or feed supplements to assess carcass quality, meat quality, growth performance, and digestibility of livestock. Few studies have been reported on the use of citrus waste (peel) on livestock performance such as broiler chickens (Ebrahimi et al., 2015; Ahaotu et al., 2017). In addition, we observed that studies on the citrus peel as waste were mainly used to assess its potential as an antimicrobial and antioxidant agent (Ahaotu et al., 2017). According to Benavente-Garcia and Castillo (2008) citrus fruits are known to possess other non-nutrient yet biologically active compounds such as flavonoids, carotenoids, vitamins, and minerals which could be medicinal in nature for animals. To bridge some knowledge gaps and to promote more unique research in livestock production, research on the use of citrus waste (peel, pulp, essential oils, and extract powder) can be extended to evaluate egg production in poultry, performance parameters of other livestock such as turkey, rabbit, and snails among others. More studies on the utilization of citrus peels to advance livestock production are also worth researching. A scientometric study accompanied by a meta-analysis or a narrative review in citrus waste and animal feed research may also be of immense benefit to the pool of knowledge in this area.

The most cited article—“Effect of heat treatment on the phenolic compounds and antioxidant capacity of citrus peel extract” was authored by Xu et al. (2007). This study was published in the *Journal of Agricultural and Food Chemistry*. In this study, the authors proposed in their findings that the application of heat to citrus peel substrate has the potential of improving the antioxidant properties of citrus waste as an antioxidant agent for animals. Other authors have also reported similar studies on the activities of citrus waste as an excellent antioxidant agent (Mandalari et al., 2007; Xu et al., 2007; Lagha-Benamrouchea and Madani, 2013). The second most cited publication “Antimicrobial activity of flavonoids extracted from bergamot (*Citrus bergamia* Risso) peel, a byproduct of the essential oil industry” was published in the *Journal of Applied Microbiology* in 2007 by Mandalari et al. In the study, the authors suggested that citrus waste (peel) may be exploited from a commercial essential oil industry as a potential source of natural antimicrobials that are active against Gram-negative bacteria. From the aforementioned studies, it may be safe to state that the use of citrus waste may further be employed in addressing research questions in other areas of scientific fields.

To date, this manuscript appears to be the first scientometric study that assessed the outputs of peer-reviewed publications on citrus waste and animal feed research at a global level. Although, we are aware that there might be some shortcomings to the current study which may include and not limited to

a. Missing publications that we might not have included in the analysis of citrus waste and animal feed or its associated words during the retrieval of data from Scopus and WOS.
b. Second, shortcomings may arise from our observations since this study did not include publications on citrus waste and animal feed research that were in non-indexed journals and thus, would not have been available in Scopus and WOS databases, such as those published in some Chinese or other non-English journals.
c. The current study might also be limited due to the exclusions of other document types including meeting abstracts, review articles, and note papers.

## Conclusions

Our scientometric analysis revealed a global increase in the use of citrus waste as the animal feed for livestock production, with greater research outputs from high-income nations when compared to low- and middle-income nations and limited collaboration with developing nations based on the Scopus and WOS databases. The less number of research publications in developing nations on the current subject matter mirrored similar happenings of outputs in other research fields. Furthermore, the current study revealed lesser research outputs on the use of citrus peels (wastes) as animal feed for livestock production when compared to the use of citrus pulps for similar reasons. Despite the appreciable progress illustrating the utilization of citrus waste as animal feed over the past 40 years, many questions remained to fully address their (citrus waste as animal feed) relevance as a standard livestock feed to curb the challenge of the ever-increasing prices of conventional feed when searching for a cheaper alternative feed resource.

## Data Availability Statement

The raw data supporting the conclusions of this article will be made available by the authors, without undue reservation.

## Author Contributions

EI: conceptualization, data curation, analysis, visualization, writing original draft, and manuscript editing. YH: logistics and supervision. All authors contributed to the article and approved the submitted version.

## Conflict of Interest

The authors declare that the research was conducted in the absence of any commercial or financial relationships that could be construed as a potential conflict of interest.

## Publisher's Note

All claims expressed in this article are solely those of the authors and do not necessarily represent those of their affiliated organizations, or those of the publisher, the editors and the reviewers. Any product that may be evaluated in this article, or claim that may be made by its manufacturer, is not guaranteed or endorsed by the publisher.
